# Supplementary figures and images for: Resveratrol Reverts Tolerance and Restores Susceptibility to Chlorhexidine and Benzalkonium in Gram-Negative Bacteria, Gram-Positive Bacteria and Yeasts
Source: Antibiotics (Basel). 2022 Jul 18;11(7):961. doi: 10.3390/antibiotics11070961 (PMC9311544; doi:10.3390/antibiotics11070961)

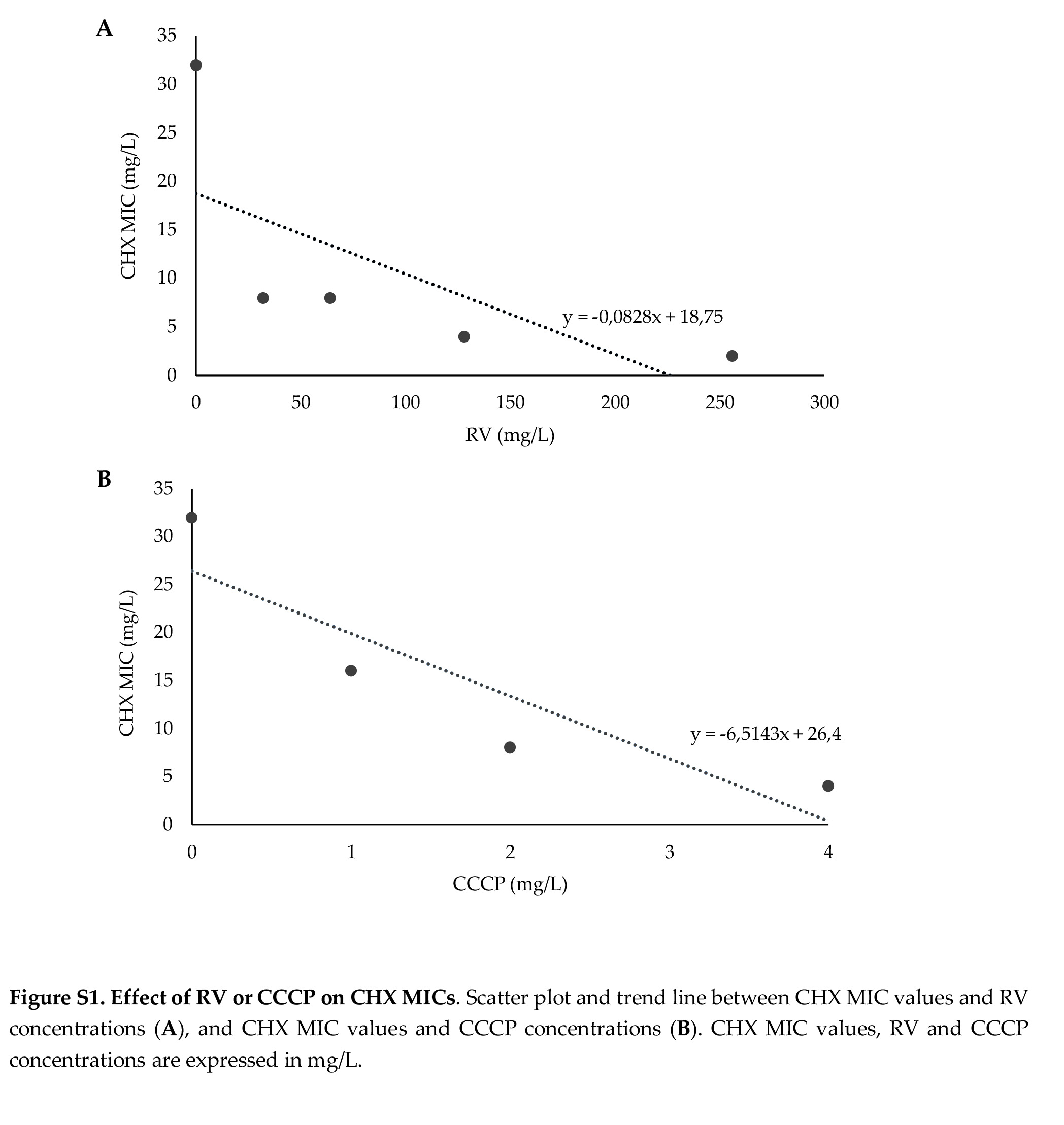

Supplement: Supplementary file 1 [file antibiotics-11-00961-s001.zip › Figure S1.jpg]

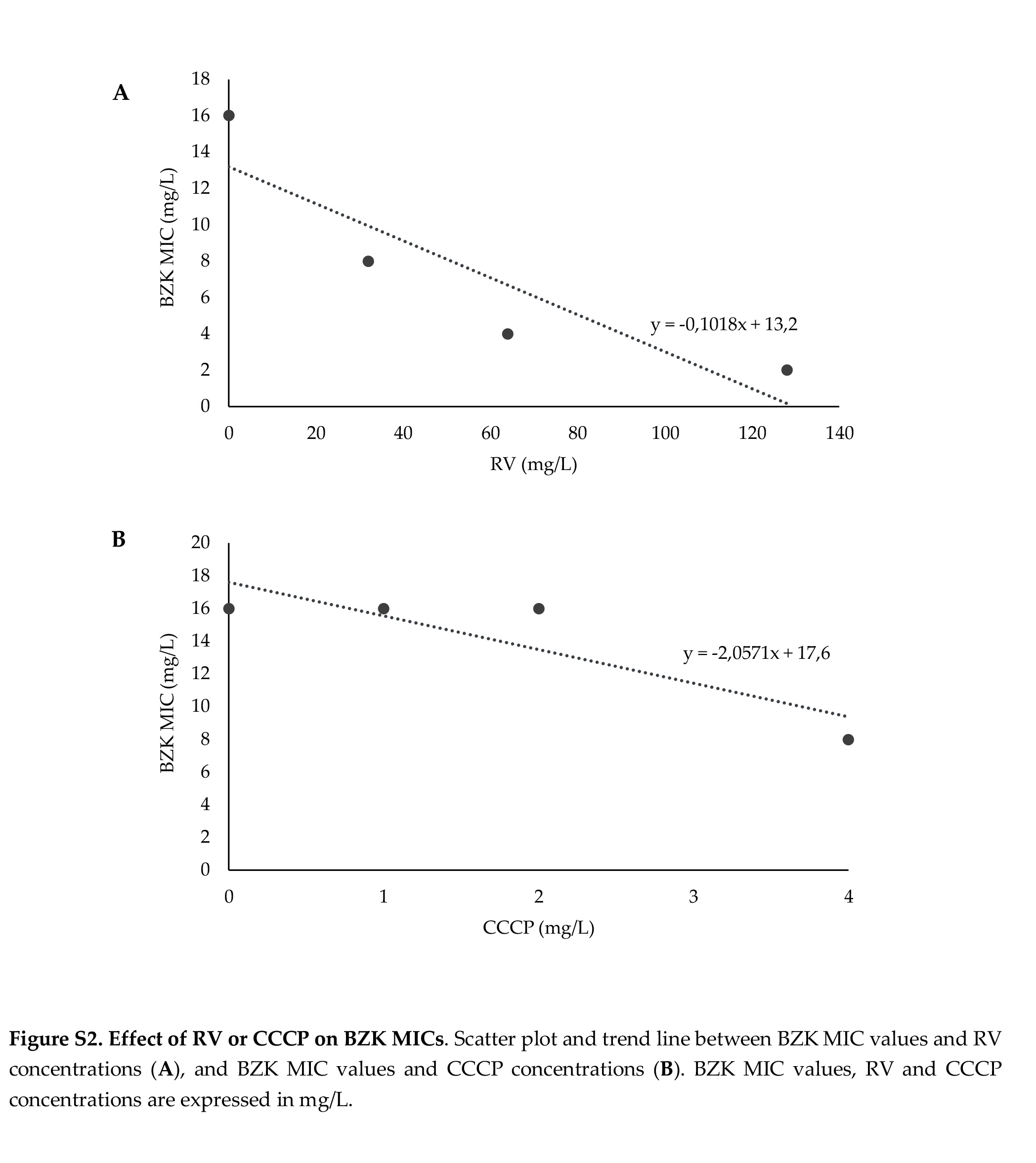

Supplement: Supplementary file 1 [file antibiotics-11-00961-s001.zip › Figure S2.jpg]
